# Challenges in enumeration of CTCs in breast cancer using techniques independent of cytokeratin expression

**DOI:** 10.1371/journal.pone.0175647

**Published:** 2017-04-19

**Authors:** John Castle, Karen Morris, Susan Pritchard, Cliona C. Kirwan

**Affiliations:** 1 Clinical and Experimental Pharmacology, Cancer Research UK Manchester Institute; Manchester, United Kingdom; 2 Department of Histopathology, University Hospital of South Manchester; Manchester, United Kingdom; 3 Division of Molecular and Clinical Cancer Sciences, University of Manchester; Department of Academic Surgery, University Hospital of South Manchester, Manchester, United Kingdom; The Ohio State University, UNITED STATES

## Abstract

**Introduction:**

Given the current postulated plasticity between epithelial and mesenchymal states of migratory cancer cells the detection of non-epithelial CTCs is an important scientific and clinical goal.

**Methods:**

We used the filtration-based ISET technology to enrich circulating tumour cells (CTCs) in early breast cancer blood samples and identify them using a morphology-based immunocytochemistry (ICC) approach.

**Results:**

We found greater numbers of putative CTCs by this approach than by the cytokeratin-based CellSearch technology, but a high number of CTC false positives were identified in healthy volunteer samples which were not reduced in successive blood draws. Preliminary work using an oestrogen receptor (ER)-based multiplex ICC method in metastatic breast cancer ISET samples indicated a low number of ER+ CTCs even at this advanced stage.

**Conclusions:**

This work highlights the challenges in enumerating CTCs without conventional epithelial markers.

## Introduction

Every year more than 50,000 women in the UK develop breast cancer, with 11,433 dying from the disease. Globally, it is the most common cause of female cancer death worldwide, with more than 500,000 women estimated to die every year from over 1.65 million cases [1, 2]. Nearly one quarter of breast cancer patients in the UK die within 10 years, with the majority presenting with distant disease several years after their initial breast cancer diagnosis. This highlights an urgent need for prognostic, predictive and pharmacodynamic biomarkers to identify patients at increased risk of recurrent disease, for early identification of recurrence, to facilitate tailored treatment and to monitor treatment response.

Circulating tumour cells (CTCs) isolated from venous blood samples could be a relatively non-invasive real-time liquid biomarker that allows detection, monitoring and phenotyping of breast cancer. CTCs are rare cells (few per 10 ml blood /100 million leukocytes and 50 billion erythrocytes), and therefore highly sensitive assays with techniques to enrich then characterise CTCs are required. Techniques to enrich for CTCs are based either on tumour cell antigen expression or cell size or density. However, all these techniques are limited by their ability to capture all CTCs given the presence of CTC heterogeneity. Once enriched, CTC identification and characterisation can be achieved on a cellular level through microscopy or flow-cytometry, or on a molecular level using RT-PCR, however once again tumour heterogeneity remains the major challenge [3].

Currently the only FDA approved CTC technology is the antibody-based CellSearch system. This identifies epithelial cells through their expression of the epithelial markers Epithelial cell adhesion molecule (EpCAM), cytokeratins 8, 18 and 19, and the lack of CD45 leukocyte marker expression. A similar, widely used technology is AdnaTest BreastCancer (AdnaGen, Langenhagen, Germany), which uses both an anti-EpCAM antibody and an antibody against the epithelial cell surface associated glycoprotein, mucin-1 (MUC-1). However CTCs are heterogeneous, and such positive selection techniques based on expression of epithelial markers are limited by the potential loss of CTC expression of such epithelial phenotype as the CTCs develop a more stem cell-like phenotype and behaviour pattern [4, 5] due to the epithelial-to-mesenchymal transition (EMT) that occurs to facilitate tumour cell adhesion, motility and subsequent intravasation.

Although CellSearch has widespread popularity in the metastatic breast cancer setting, its use in early breast cancer is more limited. A study of 2026 higher-risk early breast cancer patients (66% node positive) confirmed the presence of CTCs as an independent prognostic marker for disease-free and overall survival. However only 21.5% of patients had CTCs, despite a large blood volume (30ml) analysed [6].

In metastatic breast cancer, a major proportion of CTCs show EMT and tumour stem cell characteristics, with such characteristics being associated with treatment-resistance and worse prognosis [7]. Cytokeratin-negative CTCs may represent a more aggressive CTC subpopulation and a majority of blood-borne tumour cells [8]. Therefore whilst more established CTC enumeration and identification technologies such as CellSearch and AdnaTest employ anti-epithelial marker antibodies, more recently there has been a push towards size-based isolation to improve the CTC capture rate and better capture CTC heterogeneity. There is a range of promising non-epithelial based methodologies under evaluation, none of which have yet risen to the prominence that CellSearch has in epithelial-based CTC detection [3].

The ISET device (Rarecells) is a filtration based CTC enrichment technology that collects large blood cells (>8μm) including CTCs and circulating tumour microemboli (CTM) on a filter membrane [9]. Of the ‘large cells’ captured by this technique, CTCs are routinely identified by morphology. These CTCs and CTMs containing both epithelial and mesenchymal CTC subpopulations can then be subjected to immunomorphological, immunofluorescence, genetic, DNA or RNA analysis. ISET has allowed the identification of CTCs co-expressing cytokeratins and the mesenchymal marker vimentin [10]. Higher rates of CTC positivity by ISET than by Cellsearch using a morphology-based approach have been reported in NSCLC [11, 12], pancreatic cancer [13], metastatic prostate cancer [11] and melanoma [14] despite larger cell size criteria. This is consistent with the presence of non-epithelial CTC populations. CTC enumeration by ISET has been correlated with shorter survival in hepatocellular carcinoma [15].

In this exploratory study, we aimed to determine the role of CTC enumeration by a non-epithelial marker dependent technique, using ISET in a high-risk early (non-distant metastatic) breast cancer population, with a view to developing novel biomarkers for response to treatment.

### Patients and methods

#### Patient population

Following written informed consent, treatment-naïve, early breast cancer patients undergoing surgical resection were recruited at University Hospital of South Manchester (UHSM). Sex matched healthy controls were also recruited. CTC-positive (by CellSearch) metastatic breast cancer patients with oestrogen receptor positive disease were recruited solely for the development of an ER-based multiplex immunocytochemistry (ICC) assay.

This study was approved by Oldham Research Ethics Committee (Ref: 09/H1011/47) and NRES Committee North West—Greater Manchester Central Ethics Committee (Ref: 12/NW/0447) and sponsored by University Hospital of South Manchester.

#### Blood sampling

A 20ml sample of peripheral venous blood was collected using CellSave preservative and EDTA vacutainer tubes for CellSearch and ISET analysis respectively. Blood was collected preoperatively and at surgery shortly after tumour removal, to investigate any systemic tumour or epithelial cell release as a result of surgical tissue handling. Blood was stored at room temperature until analysis.

#### ISET filtration

Within four hours of venesection, whole blood was diluted 1:9 with Rarecells buffer, and left to stand for 10 minutes. This allowed lysis of erythrocytes and fixation of nucleated cells as previously described [9]. The mixture was then filtered using the ISET filtration device at a pressure of 5–9 kPa as per manufacturer’s instructions (Rarecells) to allow capture of large cells (>8μm) on the ISET filter membrane. The membrane was allowed to dry overnight at room temperature before storage at -20°C.

#### Immunocytochemical (ICC) staining of ISET filters: Morphology based method

Following thawing ISET ‘spots’ from filters corresponding to 1ml whole blood were rehydrated using TBS buffer and subjected to antigen retrieval in a 99°C water bath using pH6 retrieval solution (Cat# S1699, Dako). Following permeabilisation (TBS + Triton X-100) and peroxidase blocking steps, the ISET spots were incubated with a primary mouse CD45 (Monoclonal, C7230, Dako, Glostrup, Denmark, 1/30 dilution) and CD144 (Monoclonal, 14-1449-82, eBioscience, San Diego, USA, 1/50 dilution) antibody cocktail at 4°C overnight. Anti-mouse-HRP secondary antibody (K4001, Dako) was applied the next day, with DAB substrate subsequently applied and the spots counterstained with haematoxylin. Spots were mounted onto slides using Faramount mounting media and allowed to set overnight at room temperature.

#### Multiplex immunocytochemical (ICC) oestrogen receptor alpha (ERα)-based staining

The nuclear ER receptor represents a potential non-epithelial marker of CTCs in the ER positive subset of breast cancer patients. Enzo Life Science’s Multiview (Mouse-HRP/Rabbit-AP) multiplex IHC Kit (ADI-050-100-0001, New York, USA) was used in combination with a rabbit Oestrogen Receptor alpha (ERα) antibody (Monoclonal, ab108398, Abcam, Cambridge, UK, 1/100 dilution) in a method incorporating the antigen retrieval, permeabilisation, peroxidase blocking and CD45/CD144 staining steps described above. ERα/CD45/CD144-stained ISET spots were mounted onto slides using Faramount mounting media as above. Oestrogen receptor positive MCF-7 breast cancer cells (ATCC HTB-22, obtained directly) spiked into healthy donor blood were used in assay development (S1 Fig).

#### Scoring of CTCs

**Fig 1:**
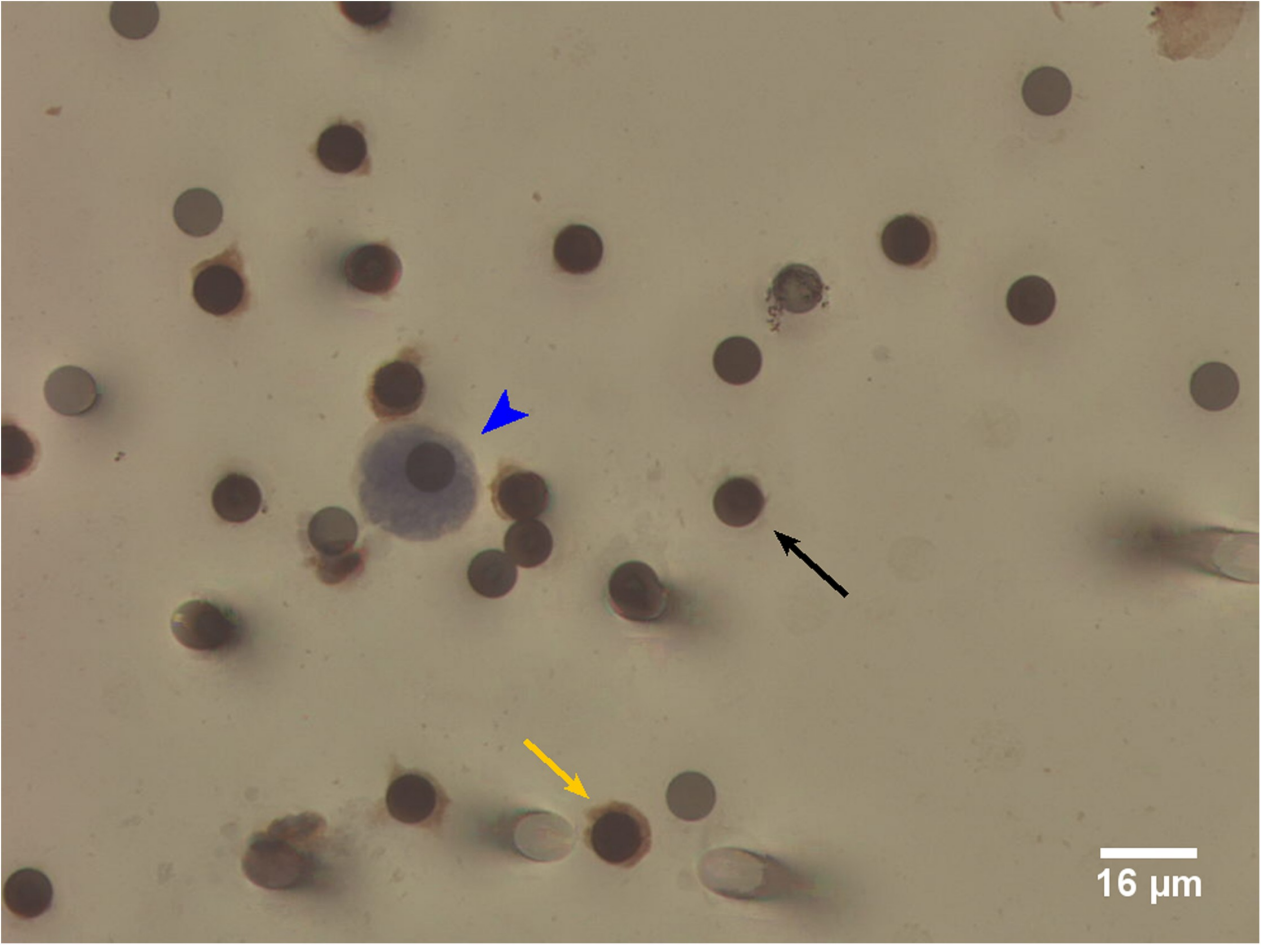
Circulating Tumour Cell (CTC) detected by Immunocytochemical (ICC)-Staining of ISET Filters. Large cells from blood samples are enriched by ISET filtration and immunocytochemically stained for the White Blood Cell marker CD45 and the Endothelial Cell marker CD144 (yellow arrow). Circulating Tumour Cells (CTCs) are identified as ≥16μm diameter cells with a hyperchromatic nucleus and negative for CD45/CD144 brown chromogen staining (blue arrowhead). The dark circles are 8μm filter pores (black arrow).

The mounted ISET spots were scanned using a Bioview automated light microscopy scanning system based on an Olympus BX61 microscope, using Duet software (Bioview, Rehovot, Israel). A circle of radius of 4000μm from the centre of each ISET spot was scanned to capture all cells. Galleries of images were manually reviewed using the Bioview Solo software. In the morphology based approach, CTCs were identified based on a set morphology definition as per previous publications [12–14] and under the guidance of a consultant breast-specialist histopathologist (SP). All images were reported by JC, with a subset of the images reviewed by SP for concordance. Cells greater or equal to 16μm diameter, with hyperchromatic nuclei and negative for CD45/CD144 brown chromogen staining were classified as CTCs (Fig 1). For the multiplex immunocytochemistry (ICC) ERα-based approach, ERα positive CTCs were defined as cells ≥16μm diameter with positive for ERα red chromogen staining and negative for CD45/CD144 brown chromogen staining. Four ISET spots corresponding to 4ml whole blood were ICC stained and scored for each blood sample. The number of CTCs detected was extrapolated to the equivalent of 7.5ml for comparison with concurrent CellSearch analysis.

#### CellSearch CTC analysis

CellSave blood samples (7.5ml) were processed by the CellSearch system as described elsewhere [16]. Briefly, CTCs are immunomagnetically separated from other blood components by EpCAM (epithelial cell adhesion molecule) antibody-conjugated beads and then stained for cytokeratins (CKs 8, 18 and 19) and CD45 in a fluorescent-based approach (S2 Fig). CTCs are defined as CK+ CD45- cells over 4μm in diameter; exact CTC diameters were not measured as the system software does not have this functionality.

## Results

### Early breast cancer patients

To maximise the chance of finding CTCs, a relatively higher risk group of early breast cancer patients were recruited. At final pathology, 75% were node positive (Table 1). Of the 16 patients, six had CTCs by CellSearch and all had CTCs by the ISET morphology-based assay at least one time-point (Table 2).

**Table 1:** Early breast cancer patient and tumour demographics. Tumour size, grade, ER (invasive and DCIS) and lymph node, HER2, Ki67 (invasive only) were determined as per National Health Service Breast Screening Programme Guidelines. Patient 8 had an invasive lobular cancer, all other patients had invasive ductal cancer. +, positive receptor status; -, negative receptor status.

**Study ID number**	**Age**	**Grade**	**Size**	**Number of nodes positive**	**ER receptor status**	**PR receptor status**	**Ki67**	**HER2 status**
**1**	82	3	18	3	-	-	59	-
**2**	45	3	40	11	-	-	68	+
**3**	68	3	26	0	-	-	80	-
**4**	55	3	40	0	+	-	61	-
**5**	39	3	60	4	+	+	31	-
**6**	69	2	18	2	+	+	27	-
**7**	35	3	29	10	+	+	48	-
**8**	51	2	22	29	+	+	10	-
**9**	79	3	36	0	-	-	76	+
**10**	76	2	40	3	-	-	10	-
**11**	52	3	16	1	+	+	30	-
**12**	69	3	31	1	-	-	23	-
**13**	63	3	19	2	-	-	45	+
**14**	73	3	30	4	+	-	33	-
**15**	74	1	19	1	+	+	23	-
**16**	33	2	21	0	+	+	18	-

**Table 2:** Enumeration of CTCs by ISET and CellSearch in early breast cancer patients before and immediately after surgery. Pre-operative blood samples were taken prior to induction of anaesthetic. Early postoperative blood samples were taken within 15 minutes of tumour removal. Late postoperative samples were taken approximately 30–60 minutes after tumour removal (Subject 2 only). *Missing sample due to inadequate blood volume or analysis failure.

**ID number**	**Pre-op sample** **(number of CTCs per 7.5ml whole blood)**		**Early post op blood sample (number of CTCs per 7.5ml whole blood)**		**Late post op blood sample (number of CTCs per 7.5ml whole blood)**	
	ISET	CellSearch	ISET	CellSearch	ISET	CellSearch
**1**	19	1	7	12		
**2**	4	1	17	0	6	0
**3**	30	1	34	2		
**4**	32	0	13	*		
**5**	37	0	*	*		
**6**	6	0	*	0		
**7**	22	0	26	1		
**8**	15	13	22	13		
**9**	21	0	2	0		
**10**	41	*	39	0		
**11**	11	1	9	0		
**12**	39	0	32	0		
**13**	21	0	49	0		
**14**	6	*	6	0		
**15**	*	0	9	0		
**16**	62	0	6	0		

**Fig 2:**
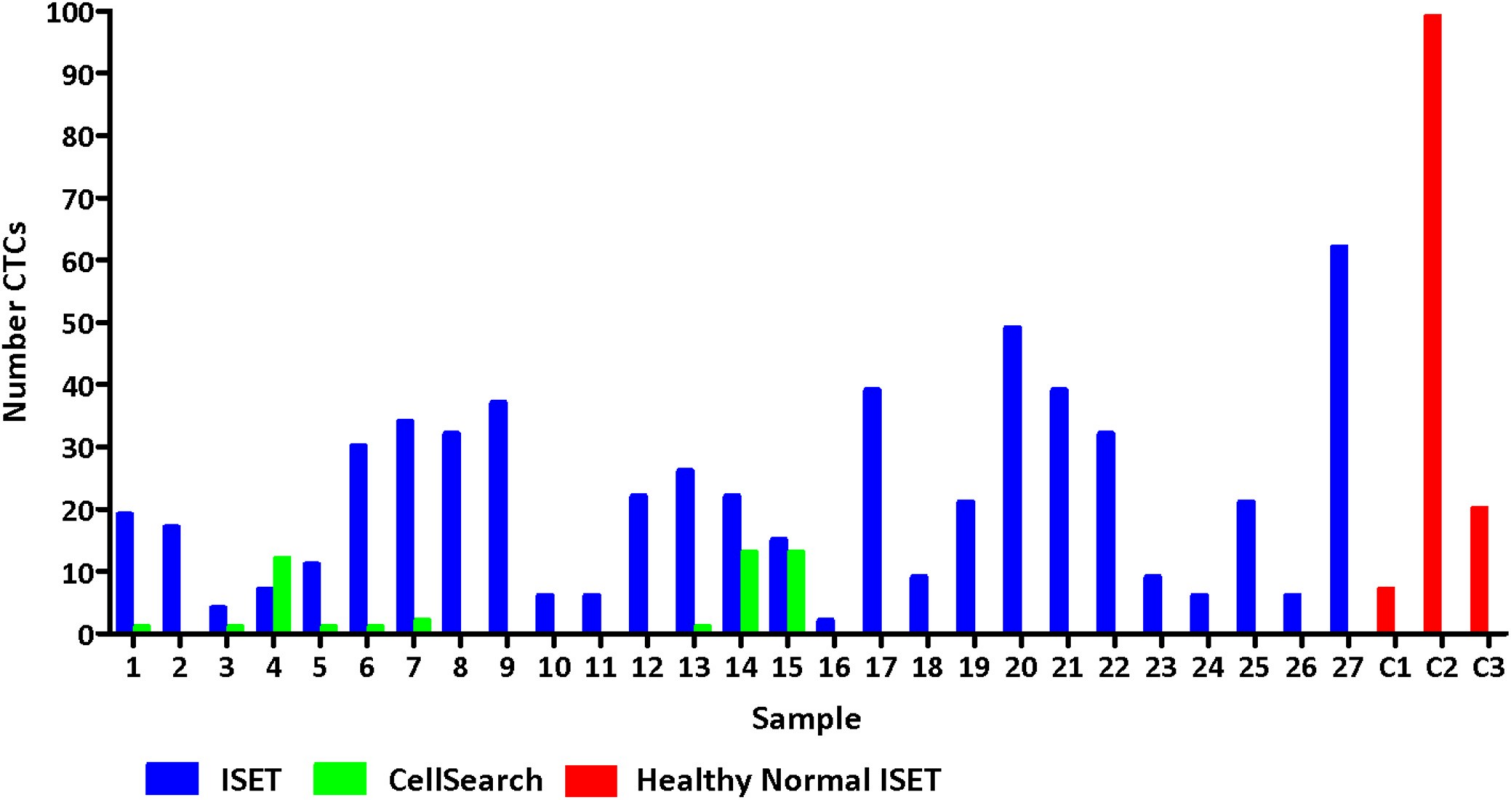
A comparison of CTC enumeration by CellSearch (based on epithelial expression) and ISET (based on morphology) in early breast cancer patients and healthy controls. Whole blood samples from early breast cancer patients and sex-matched healthy normal volunteers were analysed by both CellSearch technology (cancer patients only, green bars) and immunocytochemical (ICC) -staining of ISET filters (patients and controls, blue/red bars). The number of CTCs detected per 7.5ml whole blood is shown.

Using ISET, CTCs were detected in all 27 early breast cancer patient blood samples tested, whereas CTCs were only detected in 9 (35%) of the samples analysed by CellSearch. ISET CTC number was greater than CellSearch number in 26/27 samples (Fig 2). The median (range) number of CTCs per 7.5ml whole blood detected by CellSearch and ISET respectively were CellSearch: 0 (0–13) and ISET: 21 (2–62). The CellSearch system does not allow accurate cell size measuring so sizes of CTCs detected by each method were not compared.

There was no correlation between number of CTCs detected by ISET and detected by CellSearch, either preoperatively, postoperatively or when all sample time-points were analysed together. Number of CTCs (ISET or CellSearch) did not correlate with tumour size, node positivity or timing of blood sampling (preoperation versus postoperation); however the limited sample size is acknowledged.

The high number of CTCs found by ISET, independent of clinicopathological risk factors or CellSearch CTC numbers (although possibly reflecting an underpowered sample) raised the concern of false positive identification of CTCs by ISET.

### High CTC false positive numbers are detected in healthy volunteers by ISET morphology method

**Fig 3:**
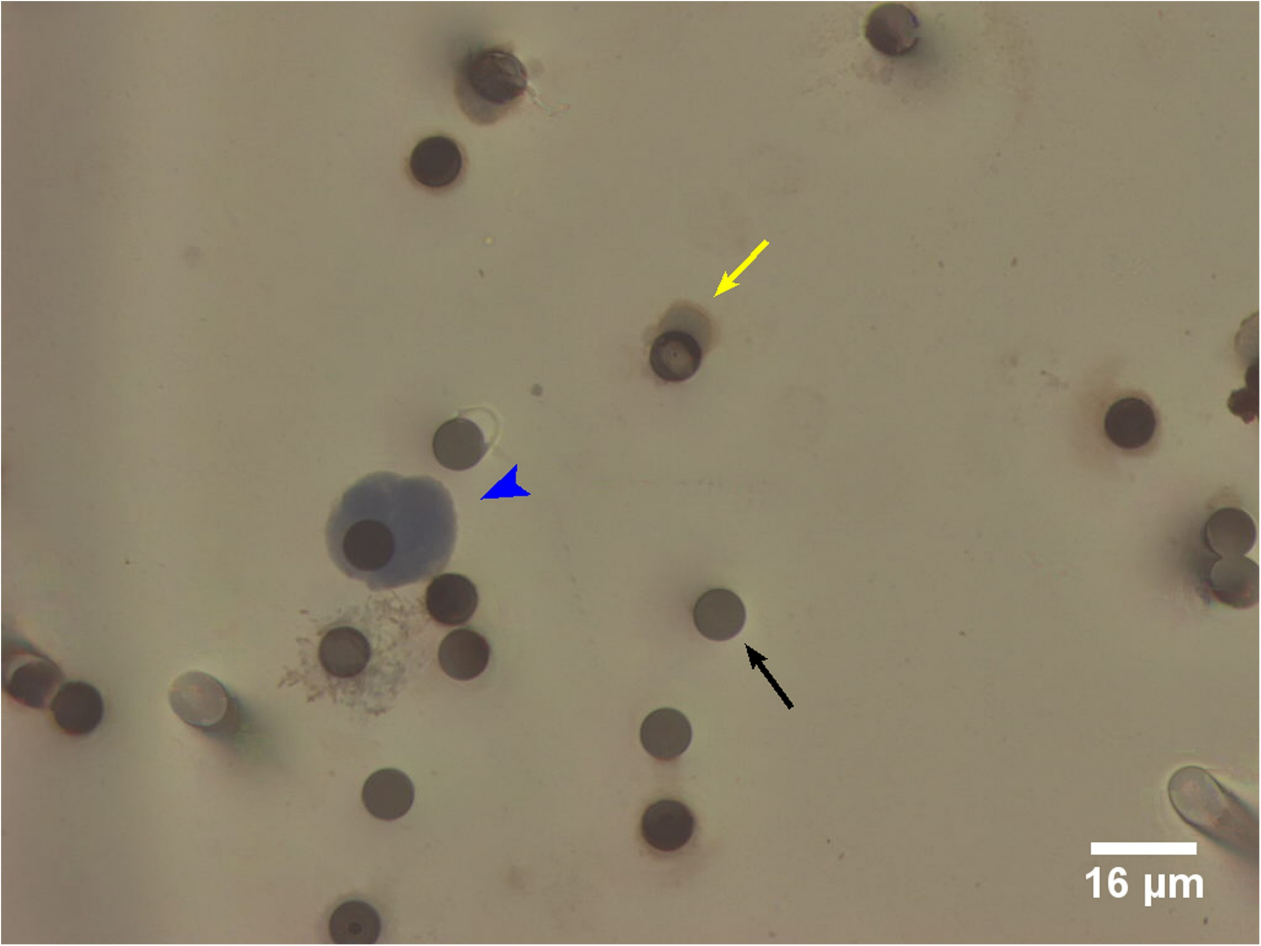
False positive CTCs identified by ISET filtration and morphology criteria in healthy volunteers. Presumed false positive ‘CTC’ (blue arrow head). Cell staining positive for CD45/CD144, consistent with white blood cell or endothelial cell (yellow arrow). The dark circles are 8μm filter pores (black arrow).

ISET filter membranes generated from three sex-matched healthy volunteer blood samples were analysed alongside the early breast cancer samples as presumed negative controls. Surprisingly, high numbers of false positive CTCs were detected, including one sample with 99 ‘CTCs’/7.5ml blood (Fig 3). These presumed false positives could not be morphologically distinguished from CTCs detected in the early breast cancer samples by the consultant breast histopathologist.

### ISET healthy volunteer CTC false positives remain high in successive blood draws

It was hypothesised that epithelial cells may be shed into the bloodstream during the first draw of venous blood collection, which could partly explain the high numbers of CTC-false positives seen in healthy volunteer samples. In three further sex-matched healthy volunteers the number of CTCs detected in first, second and third blood draws were compared (Table 3). A trend towards lower ‘CTC' numbers in successive draws was observed, but in all samples ‘CTC’ number was unacceptably high, highlighting the high false positive rate of the morphology based ISET technique.

**Table 3:** ISET ICC ‘CTCs’ in healthy normal control successive blood draws. Female healthy volunteer first, second and third draw whole blood samples were analysed by immunocytochemical (ICC) staining of ISET filters. Following venepuncture, three 10ml EDTA bottles were filled in succession from each volunteer, and analysed separately. The number of CTCs detected per 7.5ml blood is shown.

**Healthy Female Volunteer ID**	**1**^**st**^ **Draw**	**2**^**nd**^ **Draw**	**3**^**rd**^ **Draw**
**V1**	**75**	**60**	**86**
**V2**	**51**	**39**	**28**
**V3**	**103**	**41**	**41**

### Low numbers of ER+ CTCs are detected in metastatic breast cancer patients

Given the apparent high false positive CTCs identified by ISET-morphology, a similar but non-comparable ERα-based multiplex immunocytochemistry staining assay was employed with the aim of increasing specificity of CTC identification in ER positive breast cancer patients. The methodology was performed on blood taken from three ER-positive metastatic breast cancer patients. The samples from each patient were taken at the same blood draw, and processed for ISET (EDTA) and CellSearch CTC analysis. ISET samples from patients identified as CTC positive on CellSearch were examined. ERα positive CTCs were identified by ISET, however the number of CTCs identified by this methodology was markedly lower than by CellSearch, even in a high-CTC population indicating that this assay would be inappropriate for the low numbers of CTCs found in early breast cancer sample analysis (Table 4).

**Table 4:** Enumeration of CTCs in metastatic breast cancer patients by Oestrogen receptor alpha ISET filter ICC staining detects fewer CTCs than compared to the CellSearch. Whole blood samples, collected at the sample venesection, from ER positive metastatic breast cancer patients were analysed by both immunocytochemical (ICC) staining of ISET filters and by the CellSearch technology. The number of CTCs detected per 7.5ml whole blood is shown.

**Metastatic Breast Cancer Patient ID**	**CellSearch CTC Number**	**ERα+ CTCs by ISET ICC**
**1**	**10**	**3**
**2**	**16**	**0**
**3**	**4**	**1**

## Discussion

Using the ISET platform and an epithelial-marker independent immunocytochemical assay we detected higher number of putative CTCs in early breast cancer samples than CTCs detected by the EpCAM/cytokeratin dependent CellSearch. However, in an unexpected result, by the same cell morphology based technique we also found high numbers of false positive ‘CTCs’ in healthy volunteer blood samples. By employing oestrogen receptor alpha as a positive marker we developed an immunocytochemical assay which we applied to a small number of ER+ metastatic breast cancer samples, however this technique identified minimal numbers of CTCs despite evidence of their presence on CellSearch.

Much of the previously published literature on the use of the ISET device in detecting supposed CTCs using morphological criteria has found higher numbers of CTCs than are detected by epithelial antigens across a range of cancer types, despite larger size criteria [11–14, 17–19]. These studies have identified ‘CTCs’ by a range of cell and nuclear morphological criteria unlike the standard epithelial-marker based approaches (Table 5).

**Table 5:** Published studies using ISET filtration technique to capture CTCs, with a variety of morphological identification techniques. Studies identifying CTCs from patient blood samples using ISET without the use of epithelial markers. All studies utilised nuclear morphology criteria such as nuclear contour irregularity, nuclear-cytoplasmic size ratio and hyperchromasia but with varying cell/nucleus size criteria and use of the leukocyte common antigen CD45 as a negative marker. N/A, Not Applicable; ND, Not Done.

**Author (year)**	**Cancer Type(s)**	**Cell/** **Nuclear Diameter** (*, not defined)	**CD45 Negative**	**CTC positivity in cancer patients**	**CTC positivity in non-cancer patients**
**Abdallah et al. 2015 [20]**	Metastatic Colorectal Cancer	>12μm/*	Y	43/52	ND
**De Giorgi et al. 2010 [21]**	Melanoma	≥16μm/*		23/87	0/48
**De Giorgi et al. 2010 [22]**	Melanoma	≥16μm/*		N/A	N/A
**Farace et al. 2011 [11]**	Breast/Prostate/Lung	*/≥16μm		57/60	ND
**Hofman et al. 2010 [17]**	NSCLC	*/>24μm		76/208	0/39
**Hofman et al. 2010 [23]**	NSCLC	*/>24μm		102/250	0/59
**Hofman et al. 2011 [24]**	Breast/Colon/Kidney/ Head and Neck	*/>24μm		245/569	12/239
**Khoja et al. 2012 [13]**	Pancreatic	>10μm/*	Y	26/29	ND
**Krebs et al. 2012 [12]**	NSCLC	*/≥12μm	Y	32/40	ND
**Lecharpentier et al. 2011 [10]**	NSCLC	≥16μm/≥16μm		6/6	0/6
**Li et al. 2015 [25]**	Oesophageal squamous cell carcinoma	*/>18μm		20/61	0/22
**Morris et al. 2014 [19]**	Hepatocellular Carcinoma	≥16μm/*		19/19	ND
**Vona et al. 2004 [15]**	Hepatocellular Carcinoma	≥25μm/*		23/44	0/107

Vona et al. (2004) [15] in the first clinical article using the ISET technology described that CTCs/CTMs were detected in 53% of liver cancer samples by cytomorphological analysis but none were detected in 107 non-cancer patients, including many with other types of liver disease. However, the >25μm cell diameter criterion they used is in our view inappropriate for breast cancer. This size would exclude most of the breast cancer CTCs we have encountered by CellSearch and the most commonly used breast cancer cell lines [26]. Furthermore, an independent albeit smaller hepatocellular carcinoma study carried out in our laboratory concurrent to this work found no significant relationships between ISET-derived CTC number and clinical characteristics, raising doubts about the utility of identifying CTCs by morphology alone [19].

In a subgroup of twenty metastatic breast cancer patients Farace et al. used a nuclear size cut-off of ≥16μm to define CTCs in ISET, finding 17/20 patients to be CTC-positive [11]. However, no healthy subjects were examined in their study as controls. Hofman et al. in large NSCLC-focused clinical studies that included a breast cancer patient subset have developed their own ISET cytomorphology-based scoring method [23]. Identified ‘circulating non-haematological cells’ (CNHCs) were further characterised as CNHCs with malignant features (CNHC-MFs, ‘CTCs’) if they exhibited four of the following criteria: anisonucleosis, nucleus size >24μm, irregular nuclei, presence of tridimensional sheets, and a high nuclear-cytoplasmic ratio. CNHC-MFs/CTCs were identified in 43% malignant disease but only 5% of non-cancer disease patients [24]. Although inter-observer agreement for detection of CNHCs was an impressive 100% (κ = 1) between three assessing cytopathologists, it is noteworthy that inter-observer agreement of CNHCs with benign features was relatively low (κ = 0.35). El-Hilibi et al. replicated their methodology in an alternative filtration based technology (ScreenCell) and determined that morphological criteria alone were inadequate to distinguish malignant from non-malignant cells [27].

As CellSearch cell size criteria is >4μm, compared to our ISET definition of ≥16μm, it might be expected that CellSearch would identify higher CTC numbers. However, like several other authors, we found higher numbers with ISET [11–14, 17–19]. Although many presume this is because of identification of non-epithelial antigen expressing CTCs, CTC false positivity is an acknowledged problem in filtration based technologies [28]. This is supported by our high CTC identification in normal controls. ISET-based studies have used large cell/nuclear size criteria to avoid misidentification of endogenous nucleated blood cells at the expense of identifying smaller CTCs (Table 5). Small CTCs (≤90 μm^2^) have been described in prostate cancer [29] with small-nuclear CTCs being associated with visceral metastases [30], and therefore may represent an important subgroup where the ISET technology produces false negatives. There are several possible causes of the false positives seen in this study. It is possible that larger leukocytes may have been misidentified, especially monocytes which can reach up to 20μm in diameter [31], although the CD45 immunocytochemistry method employed has previously been shown to be effective at staining these cells [12]. Another candidate is megakaryocytes, responsible for platelet production with a 37μm mean cell diameter however these are usually discounted due to their round and pale nucleus and high reported CD45 expression [32]. Epithelial cells, which can be collected during peripheral venous blood collection by intradermal needle could also have been present on the filters and misidentified as CTCs [33]. In addition, El-Hilibi et al. suggested circulating endothelial cells as a probable cause of misidentified CTCs in their study [27]. Endothelial cells are released during venepuncture [34], with numbers shown to decline on subsequent draws [35]. This prompted us to examine ‘CTC’ numbers in successive blood draws as shown in *Table 3*. Although there was a trend of lower false positives in each subsequent draw, the high numbers seen in the third draw indicates that this is not the sole cause of this phenomenon.

Also, by using VE-Cadherin, CD144 as a negative stain, we have gone further than the studies in *Table 5*in actively identifying endothelial cells using a specific protein marker. Platelet endothelial cell adhesion molecule-1 (PECAM-1/CD31) as a further endothelial marker and CD61 as a marker for megakaryocytes may provide additional support in correctly identifying CTCs [36, 37]. However, approximately 90% of all putative CTCs we observed on stained ISET filters created by standard device operation were sucked into the 8μm pores, which plainly affected their cell and nuclear morphology. This factor undoubtedly contributed to the high rate of false positives seen. Based on these results, morphology based ISET CTC enumeration is not appropriate for early breast cancer.

Oestrogen receptor alpha (ERα) expression in the primary tumour is routinely assessed in breast cancer clinical management [38]. Clinical studies have previously examined ERα expression in cytokeratin/EpCAM positive CTCs [39, 40], but to our knowledge we are the first to identify CTCs primarily by using ERα as a positive marker. The lower cell count found by ISET compared to CellSearch may reflect loss of ERα expression by CTCs. This is supported by Babayan et al. [41] who stated that CTCs frequently lack ER expression in ER+ metastatic breast cancer. Atkas et al. (2011) have also reported discordance between CTC and tumour ER expression [42]. However the lower CTC numbers reported by ISET compared to CellSearch when ERα expression is included may possibly be a result of ISET failing to capture smaller ‘true’ CTCs. This further highlights the one of the challenges of the ISET technology. In addition, the number of ISET ERα positive identified CTCs in the setting of metastatic disease was surprisingly low compared to ISET ‘CTCs’ identified by just morphology in the early breast cancer subgroup, as it would be expected that CTC counts would be higher in more advanced disease. Our finding of low CTC numbers in metastatic disease compared to early disease further highlights the likely presence of false positives with the morphology-only technique used in the early breast cancer group.

One of the particular challenges we encountered with this methodology is the time required for blood analysis. To ensure the integrity of nucleated cells, whole blood samples in EDTA vacutainers must be diluted in Rarecells buffer within four hours of blood taking, presenting challenges in sample transport and analyst availability. Blood processing and filtration using the ISET technology is non-automated and requires an average of 60 minutes of technician time per sample. However, even more challenging, the images produced from each spot corresponding to 1ml whole blood (up to 10 spots per sample) require 30 minutes to view and score, highlighting the limitations of this technology in its current form.

In summary, we investigated the use of the ISET technology to enumerate CTCs by a non-cytokeratin / epithelial marker technique in early breast cancer patients. The high false positive rates based on morphology alone, and the low numbers captured (presumed high false negative) by ERα plus morphology raises doubts on its utility in this low burden disease. This study highlights the need for caution in the use of all non-cytokeratin based CTC enumeration methodologies, with a necessity for extensive examination of normal control samples. However, considering the current postulated plasticity between epithelial and mesenchymal states of migratory cancer cells [43] the detection of non-epithelial CTCs remains an important scientific and clinical goal.

## Supporting information

S1 Fig MCF-7 cells in a spiked whole blood sample following ISET Filtration and identification by morphology and oestrogen receptor immunocytochemistry (ICC) staining. MCF-7 breast cancer cells spiked into whole blood can be identified by the red chromogen staining (red arrow). The Leukocyte Common Antigen CD45 and the Endothelial Cell marker CD144 provided counterstains to allow identification of non CTC large cells (stained brown). The dark circles are 8μm filter pores (black arrow).
(TIF)

S2 Fig CTC scoring by the CellSearch Technology uses epithelial criteria. Figure represents the gallery of images shown on the Cellsearch Analyzer after CTC enrichment from blood using EpCAM antibodies and staining. Cells ≥4μm immunofluorescently staining for cytokeratins and not CD45 are scored as CTCs. The event shown was scored as a CTC by trained analysts.
(TIF)

## References

[1] Cancer Research UK Breast Cancer Statistics http://www.cancerresearchuk.org/health-professional/cancer-statistics/statistics-by-cancer-type/breast-cancer#heading-Two Accessed September 2016.

[2] Cancer Research UK Breast Cancer Statistics http://www.cancerresearchuk.org/health-professional/cancer-statistics/statistics-by-cancer-type/breast-cancer/mortality Accessed September 2016.

[3] Castle J, Shaker H, Morris K, Tugwood JD, Kirwan CC. The significance of circulating tumour cells in breast cancer: a review. Breast. 2014 10;23(5):552–60. doi: 10.1016/j.breast.2014.07.002 25124235 10.1016/j.breast.2014.07.002

[4] Biggers B, Knox S, Grant M, Kuhn J, Nemunatitis J, Fisher T, et al Circulating tumor cells in patients undergoing surgery for primary breast cancer: preliminary results of a pilot study. Annals of surgical oncology. 2009 4;16(4):969–71. doi: 10.1245/s10434-009-0314-y 19190965 10.1245/s10434-009-0314-y

[5] Battula VL, Evans KW, Hollier BG, Shi Y, Marini FC, Ayyanan A, et al Epithelial-mesenchymal transition-derived cells exhibit multilineage differentiation potential similar to mesenchymal stem cells. Stem cells. 2010 8;28(8):1435–45. Pubmed Central PMCID: 3523728. doi: 10.1002/stem.467 20572012 10.1002/stem.467 PMC3523728

[6] Rack B, Schindlbeck C, Juckstock J, Andergassen U, Hepp P, Zwingers T, et al Circulating tumor cells predict survival in early average-to-high risk breast cancer patients. Journal of the National Cancer Institute. 2014 5;106(5). Pubmed Central PMCID: 4112925. 10.1093/jnci/dju066 PMC4112925 24832787

[7] Aktas B, Tewes M, Fehm T, Hauch S, Kimmig R, Kasimir-Bauer S. Stem cell and epithelial-mesenchymal transition markers are frequently overexpressed in circulating tumor cells of metastatic breast cancer patients. Breast cancer research: BCR. 2009;11(4):R46 Pubmed Central PMCID: 2750105. doi: 10.1186/bcr2333 19589136 10.1186/bcr2333 PMC2750105

[8] Krawczyk N, Banys M, Hartkopf A, Hagenbeck C, Melcher C, Fehm T. Circulating tumour cells in breast cancer. Ecancermedicalscience. 2013;7:352 Pubmed Central PMCID: 3776645. doi: 10.3332/ecancer.2013.352 24066018 10.3332/ecancer.2013.352 PMC3776645

[9] Vona G, Sabile A, Louha M, Sitruk V, Romana S, Schutze K, et al Isolation by size of epithelial tumor cells: a new method for the immunomorphological and molecular characterization of circulatingtumor cells. The American journal of pathology. 2000 1;156(1):57–63. Pubmed Central PMCID: 1868645. doi: 10.1016/S0002-9440(10)64706-2 10623654 10.1016/S0002-9440(10)64706-2 PMC1868645

[10] Lecharpentier A, Vielh P, Perez-Moreno P, Planchard D, Soria JC, Farace F. Detection of circulating tumour cells with a hybrid (epithelial/mesenchymal) phenotype in patients with metastatic non-small cell lung cancer. British journal of cancer. 2011 10 25;105(9):1338–41. Pubmed Central PMCID: 3241564. doi: 10.1038/bjc.2011.405 21970878 10.1038/bjc.2011.405 PMC3241564

[11] Farace F, Massard C, Vimond N, Drusch F, Jacques N, Billiot F, et al A direct comparison of CellSearch and ISET for circulating tumour-cell detection in patients with metastatic carcinomas. British journal of cancer. 2011 9 6;105(6):847–53. Pubmed Central PMCID: 3171010. doi: 10.1038/bjc.2011.294 21829190 10.1038/bjc.2011.294 PMC3171010

[12] Krebs MG, Hou JM, Sloane R, Lancashire L, Priest L, Nonaka D, et al Analysis of circulating tumor cells in patients with non-small cell lung cancer using epithelial marker-dependent and -independent approaches. Journal of thoracic oncology: official publication of the International Association for the Study of Lung Cancer. 2012 2;7(2):306–15. 10.1097/JTO.0b013e31823c5c16 22173704

[13] Khoja L, Backen A, Sloane R, Menasce L, Ryder D, Krebs M, et al A pilot study to explore circulating tumour cells in pancreatic cancer as a novel biomarker. British journal of cancer. 2012 1 31;106(3):508–16. Pubmed Central PMCID: 3273340. doi: 10.1038/bjc.2011.545 22187035 10.1038/bjc.2011.545 PMC3273340

[14] Khoja L, Shenjere P, Hodgson C, Hodgetts J, Clack G, Hughes A, et al Prevalence and heterogeneity of circulating tumour cells in metastatic cutaneous melanoma. Melanoma research. 2014 2;24(1):40–6. doi: 10.1097/CMR.0000000000000025 24201293 10.1097/CMR.0000000000000025

[15] Vona G, Estepa L, Beroud C, Damotte D, Capron F, Nalpas B, et al Impact of cytomorphological detection of circulating tumor cells in patients with liver cancer. Hepatology. 2004 3;39(3):792–7. doi: 10.1002/hep.20091 14999698 10.1002/hep.20091

[16] Cristofanilli M, Budd GT, Ellis MJ, Stopeck A, Matera J, Miller MC, et al Circulating tumor cells, disease progression, and survival in metastatic breast cancer. The New England journal of medicine. 2004 8 19;351(8):781–91. doi: 10.1056/NEJMoa040766 15317891 10.1056/NEJMoa040766

[17] Hofman V, Ilie MI, Long E, Selva E, Bonnetaud C, Molina T, et al Detection of circulating tumor cells as a prognostic factor in patients undergoing radical surgery for non-small-cell lung carcinoma: comparison of the efficacy of the CellSearch Assay and the isolation by size of epithelial tumor cell method. International journal of cancer. 2011 10 1;129(7):1651–60. doi: 10.1002/ijc.25819 21128227 10.1002/ijc.25819

[18] Massard C, Oulhen M, Le Moulec S, Auger N, Foulon S, Abou-Lovergne A, et al Phenotypic and genetic heterogeneity of tumor tissue and circulating tumor cells in patients with metastatic castrationresistant prostate cancer: a report from the PETRUS prospective study. Oncotarget. 2016 7 4. 10.18632/oncotarget.10396 PMC5342402 27391263

[19] Morris KL, Tugwood JD, Khoja L, Lancashire M, Sloane R, Burt D, et al Circulating biomarkers in hepatocellular carcinoma. Cancer chemotherapy and pharmacology. 2014 8;74(2):323–32. doi: 10.1007/s00280-014-2508-7 24923562 10.1007/s00280-014-2508-7

[20] Abdallah EA, Fanelli MF, Buim ME, Machado Netto MC, Gasparini Junior JL, Souza ESV, et al Thymidylate synthase expression in circulating tumor cells: a new tool to predict 5-fluorouracil resistance in metastatic colorectal cancer patients. International journal of cancer. 2015 9 15;137(6):1397–405. doi: 10.1002/ijc.29495 25721610 10.1002/ijc.29495 PMC6680263

[21] De Giorgi V, Pinzani P, Salvianti F, Panelos J, Paglierani M, Janowska A, et al Application of a filtration- and isolation-by-size technique for the detection of circulating tumor cells in cutaneous melanoma. The Journal of investigative dermatology. 2010 10;130(10):2440–7. doi: 10.1038/jid.2010.141 20535130 10.1038/jid.2010.141

[22] De Giorgi V, Pinzani P, Salvianti F, Grazzini M, Orlando C, Lotti T, et al Circulating benign nevus cells detected by ISET technique: warning for melanoma molecular diagnosis. Archives of dermatology. 2010 10;146(10):1120–4. doi: 10.1001/archdermatol.2010.264 20956643 10.1001/archdermatol.2010.264

[23] Hofman V, Long E, Ilie M, Bonnetaud C, Vignaud JM, Flejou JF, et al Morphological analysis of circulating tumour cells in patients undergoing surgery for non-small cell lung carcinoma using the isolation by size of epithelial tumour cell (ISET) method. Cytopathology: official journal of the British Society for Clinical Cytology. 2012 2;23(1):30–8. 21210876 10.1111/j.1365-2303.2010.00835.x

[24] Hofman VJ, Ilie MI, Bonnetaud C, Selva E, Long E, Molina T, et al Cytopathologic detection of circulating tumor cells using the isolation by size of epithelial tumor cell method: promises and pitfalls. American journal of clinical pathology. 2011 1;135(1):146–56. doi: 10.1309/AJCP9X8OZBEIQVVI 21173137 10.1309/AJCP9X8OZBEIQVVI

[25] Li H, Song P, Zou B, Liu M, Cui K, Zhou P, et al Circulating Tumor Cell Analyses in Patients With Esophageal Squamous Cell Carcinoma Using Epithelial Marker-Dependent and -Independent Approaches. Medicine. 2015 9;94(38):e1565 Pubmed Central PMCID: 4635756. doi: 10.1097/MD.0000000000001565 26402816 10.1097/MD.0000000000001565 PMC4635756

[26] Adams DL, Zhu P, Makarova OV, Martin SS, Charpentier M, Chumsri S, et al The systematic study of circulating tumor cell isolation using lithographic microfilters. RSC advances. 2014;9:4334–42. Pubmed Central PMCID: 4299665. doi: 10.1039/C3RA46839A 25614802 10.1039/C3RA46839A PMC4299665

[27] El-Heliebi A, Kroneis T, Zohrer E, Haybaeck J, Fischereder K, Kampel-Kettner K, et al Are morphological criteria sufficient for the identification of circulating tumor cells in renal cancer? Journal of translational medicine. 2013;11:214 Pubmed Central PMCID: 3848446. doi: 10.1186/1479-5876-11-214 24044779 10.1186/1479-5876-11-214 PMC3848446

[28] Hong B, Zu Y. Detecting circulating tumor cells: current challenges and new trends. Theranostics. 2013;3(6):377–94. Pubmed Central PMCID: 3677409. doi: 10.7150/thno.5195 23781285 10.7150/thno.5195 PMC3677409

[29] McDaniel AS, Ferraldeschi R, Krupa R, Landers M, Graf R, Louw J, et al Phenotypic diversity of circulating tumour cells in patients with metastatic castration-resistant prostate cancer. BJU international. 2016 8 18. 10.1111/bju.13631 PMC5316381 27539393

[30] Chen JF, Ho H, Lichterman J, Lu YT, Zhang Y, Garcia MA, et al Subclassification of prostate cancer circulating tumor cells by nuclear size reveals very small nuclear circulating tumor cells in patients with visceral metastases. Cancer. 2015 9 15;121(18):3240–51. Pubmed Central PMCID: 4560974. doi: 10.1002/cncr.29455 25975562 10.1002/cncr.29455 PMC4560974

[31] Leeds University Blood Histology Guide http://www.histology.leeds.ac.uk/blood/blood_wbc.php Accessed September 2016.

[32] Tomer A. Human marrow megakaryocyte differentiation: multiparameter correlative analysis identifies von Willebrand factor as a sensitive and distinctive marker for early (2N and 4N) megakaryocytes. Blood. 2004 11 1;104(9):2722–7. doi: 10.1182/blood-2004-02-0769 15198950 10.1182/blood-2004-02-0769

[33] Bain BJ. Blood Cells: A Practical Guide. Fourth Edition ed: Blackwell Publishing Ltd; 2006 2006.

[34] Strijbos MH, Verhoef C, Gratama JW, Sleijfer S. On the origin of (CD105+) circulating endothelial cells. Thrombosis and haemostasis. 2009 8;102(2):347–51. doi: 10.1160/TH08-11-0762 19652886 10.1160/TH08-11-0762

[35] Boos CJ, Lane DA, Kang D, Goon PK, Blann AD, Lip GY. Temporal and venepuncture-related decline in circulating endothelial cell capture from mixed venous blood. Journal of thrombosis and thrombolysis. 2006 10;22(2):125–31. doi: 10.1007/s11239-006-8422-z 17008979 10.1007/s11239-006-8422-z

[36] Lau AH, Lai HK, Yeung BH, Leung SL, Tsang SY, Wong YH, et al Prostacyclin receptor-dependent inhibition of human erythroleukemia cell differentiation is STAT3-dependent. Prostaglandins, leukotrienes, and essential fatty acids. 2012 3;86(3):119–26. doi: 10.1016/j.plefa.2011.12.002 22336225 10.1016/j.plefa.2011.12.002

[37] Mao SZ, Ye X, Liu G, Song D, Liu SF. Resident Endothelial Cells and Endothelial Progenitor Cells Restore Endothelial Barrier Function After Inflammatory Lung Injury. Arteriosclerosis, thrombosis, and vascular biology. 2015 7;35(7):1635–44. Pubmed Central PMCID: 4483164. doi: 10.1161/ATVBAHA.115.305519 25977568 10.1161/ATVBAHA.115.305519 PMC4483164

[38] Weigel MT, Dowsett M. Current and emerging biomarkers in breast cancer: prognosis and prediction. Endocrine-related cancer. 2010 12;17(4):R245–62. doi: 10.1677/ERC-10-0136 20647302 10.1677/ERC-10-0136

[39] Frithiof H, Welinder C, Larsson AM, Ryden L, Aaltonen K. A novel method for downstream characterization of breast cancer circulating tumor cells following CellSearch isolation. Journal of translational medicine. 2015;13:126 Pubmed Central PMCID: 4409738. doi: 10.1186/s12967-015-0493-1 25896421 10.1186/s12967-015-0493-1 PMC4409738

[40] Kalinsky K, Mayer JA, Xu X, Pham T, Wong KL, Villarin E, et al Correlation of hormone receptor status between circulating tumor cells, primary tumor, and metastasis in breast cancer patients. Clinical & translational oncology: official publication of the Federation of Spanish Oncology Societies and of the National Cancer Institute of Mexico. 2015 7;17(7):539–46. Pubmed Central PMCID: 4497875. 10.1007/s12094-015-1275-1 PMC4497875 25613123

[41] Babayan A, Hannemann J, Spotter J, Muller V, Pantel K, Joosse SA. Heterogeneity of estrogen receptor expression in circulating tumor cells from metastatic breast cancer patients. PloS one. 2013;8(9):e75038 Pubmed Central PMCID: 3776726. doi: 10.1371/journal.pone.0075038 24058649 10.1371/journal.pone.0075038 PMC3776726

[42] Aktas B, Muller V, Tewes M, Zeitz J, Kasimir-Bauer S, Loehberg CR, et al Comparison of estrogen and progesterone receptor status of circulating tumor cells and the primary tumor in metastatic breast cancer patients. Gynecologic oncology. 2011 8;122(2):356–60. doi: 10.1016/j.ygyno.2011.04.039 21605893 10.1016/j.ygyno.2011.04.039

[43] Beerling E, Seinstra D, de Wit E, Kester L, van der Velden D, Maynard C, et al Plasticity between Epithelial and Mesenchymal States Unlinks EMT from Metastasis-Enhancing Stem Cell Capacity. Cell reports. 2016 3 15;14(10):2281–8. Pubmed Central PMCID: 4802222. doi: 10.1016/j.celrep.2016.02.034 26947068 10.1016/j.celrep.2016.02.034 PMC4802222

