# Supplementary figures and images for: Challenges in enumeration of CTCs in breast cancer using techniques independent of cytokeratin expression
Source: PLoS One. 2017 Apr 19;12(4):e0175647. doi: 10.1371/journal.pone.0175647 (PMC5397021; doi:10.1371/journal.pone.0175647)

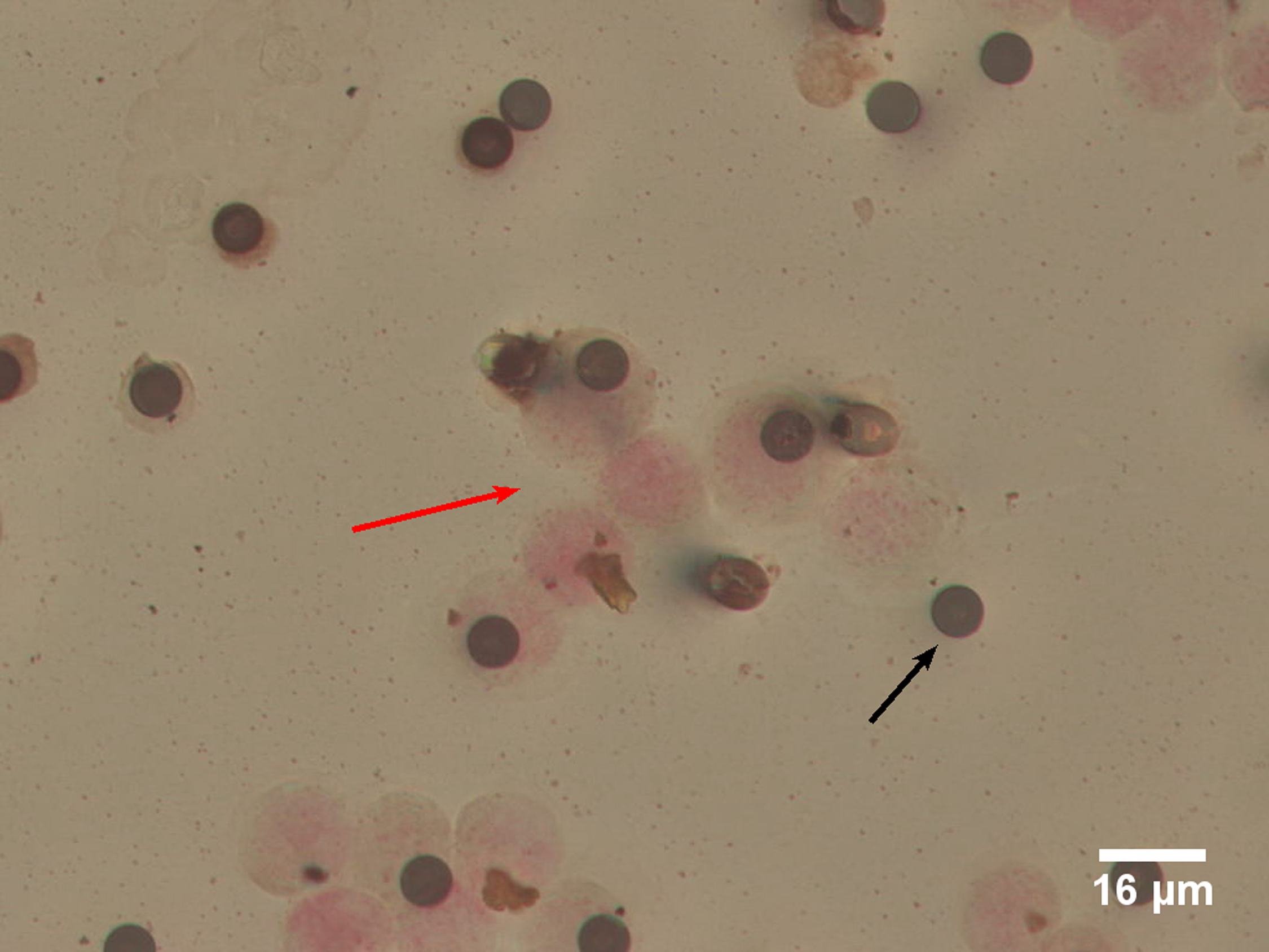

Supplement: S1 Fig — MCF-7 breast cancer cells spiked into whole blood can be identified by the red chromogen staining (red arrow). The Leukocyte Common Antigen CD45 and the Endothelial Cell marker CD144 provided counterstains to allow identification of non CTC large cells (stained brown). The dark circles are 8μm filter pores (black arrow). (TIF) [file pone.0175647.s001.tif]

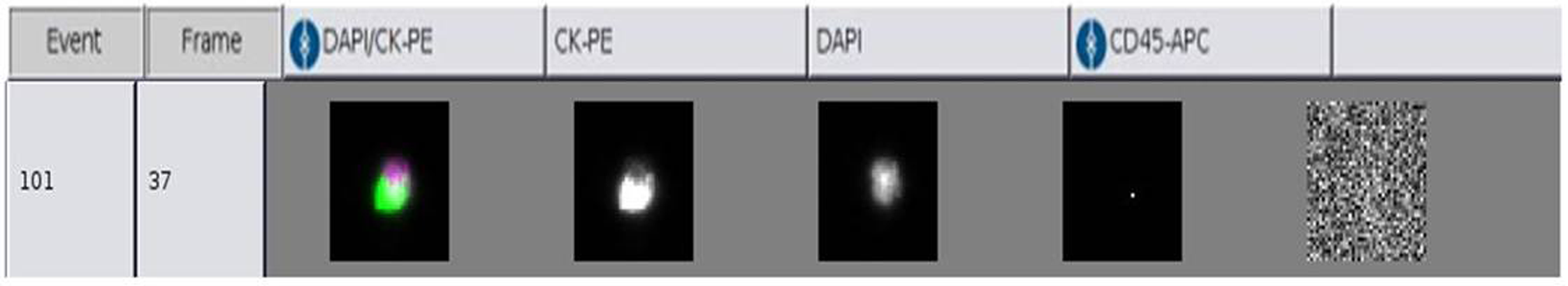

Supplement: S2 Fig — Figure represents the gallery of images shown on the Cellsearch Analyzer after CTC enrichment from blood using EpCAM antibodies and staining. Cells ≥4μm immunofluorescently staining for cytokeratins and not CD45 are scored as CTCs. The event shown was scored as a CTC by trained analysts. (TIF) [file pone.0175647.s002.tif]
